# Adhesion of individually formed fiber post adhesively luted with flowable short fiber composite

**DOI:** 10.1080/26415275.2023.2209593

**Published:** 2023-05-11

**Authors:** Anton O. Suni, Lippo V. J. Lassila, Jarno K. Tuokko, Sufyan Garoushi, Pekka K. Vallittu

**Affiliations:** a Department of Biomaterials Science and Turku Clinical Biomaterial Center (TCBC), Institute of Dentistry, University of Turku, Turku, Finland; b Wellbeing Services County of South-West Finland, Turku, Finland

**Keywords:** Short fiber composite, FRC post, luting material, push-out test

## Abstract

This laboratory study aimed to measure the push-out bond strength of individually formed fiber-reinforced composite (FRC) post luted with flowable short fiber-reinforced composite (SFRC) and to evaluate the influence of post coating with light-cured adhesive. Post spaces (Ø 1.7 mm) were drilled into 20 single-rooted decoronated premolar teeth. Post spaces were etched and treated with light-cured universal adhesive (G-Premio Bond). Individually formed FRC posts (Ø 1.5 mm, everStick) were luted either with light-cured SFRC (everX Flow) or conventional particulate-filled (PFC) dual-cure luting cement (G-CEM LinkForce). Half of the posts from each group were treated with dimethacrylate adhesive resin (Stick Resin) for 5 min before luting. After storage in water for two days, the roots were sectioned into 2 mm thick disks (*n* = 10/per group). Then, a push-out test-setup was used in a universal testing machine to measure the bond strength between post and dentin. The interface between post and SFRC was inspected using optical and scanning electron microscopy (SEM). Data were statistically analyzed using analysis of variance ANOVA (*p* = .05). Higher bond strength values (*p* < .05) were obtained when flowable SFRC was used as a post luting material. Resin coating of a post showed no significant effect (*p* > .05) on bond strength values. Light microscope images showed the ability of discontinuous short fibers in SFRC to penetrate into FRC posts. The use of flowable SFRC as luting material with individually formed FRC posts proved to be a promising method to improve the interface adhesion.

## Introduction

In recent decades, adhesive dentistry has grown quickly. Since the use of modern dental materials produces results that are superior to those of conventional ones, a number of novel and creative treatments have replaced traditional treatment approaches. The development of fiber-reinforced composite (FRC) posts as a reliable substitute for prefabricated metal posts marked a turning point in the field of dentistry [1,2]. Their modulus of elasticity being similar to dentin and their ability to bond to luting cement and tooth structure have been suggested to reduce the likelihood of root fractures most commonly associated with endodontically treated teeth (ETT) restored with metal posts [3]. This coupled with their superior esthetics and easy retrieval adds to their many advantages, making fiber-reinforced posts the material of choice in routine dental practice.

Despite the positive biomechanical behavior of FRC posts, the drawbacks have also been discussed [4–10]. The most common types of failure reported on ETT restored with adhesively luted FRC posts, were post fracture and debonding of the post. Debonding has been shown to take place at different interfaces; the cement-dentine interface; cement-post interface; and/or the composite core-post interface [4–10]. Poor bonding between a cross-linked prefabricated FRC post and luting cement and/or core material may eventually lead to marginal failure and subsequently result in secondary caries [9]. Research literature has shown that prefabricated FRC posts, compared to individually formed posts, have lower bond strength [11,12]. Improving bonding between FRC posts, luting cement and teeth increases load transfer from crown to root, which improves the restoration’s longevity [13]. In fact, large and uneven root canal spaces can be filled more effectively using an individually formed FRC post approach than with a single, prefabricated post placed in the middle of the cavity [8]. The available literature on bond strength of FRC posts is abundant, but with contradictory outcomes. This can be as a result of variations in the testing procedures, post-surface pretreatment techniques and materials employed [14,15]. However, the fact that individually formed FRC posts lack radiopacity is considered a major drawback of this material [15].

According to laboratory research [16–18], high loads can be placed on luting cements, especially in the cervical region, and *in vitro* fatigue studies have revealed that post-luting cement microfractures or cracks are the first failure mode that contributes to the development of catastrophic failure [19,20]. On the other hand, previous studies have reported that flowable short fiber-reinforced composite (SFRC) improved the load-bearing capacity of restorations when it was used as post-luting and core build-up material [21–23]. According to them, the drawbacks of using a weak link between FRC post and root dentin were apparently minimized by the tight adaptation of SFRC [21]. However, still the question arises whether the light-cured flowable SFRC material inside the root canal would have adequate bond with individually formed FRC posts.

Consequently, the purpose of this *in vitro* investigation was to verify if using flowable SFRC as a luting cement would improve the adhesion of individually formed FRC posts and therefore increase the posts’ longevity. In addition, to study the influence of FRC post coating with light-cured bonding resin on adhesion.

The null hypotheses were that the luting material type (I) and post coating (II) will have no effect on the adhesion of FRC post to dentin.

## Materials and methods

### Specimen preparation

A 20 human caries-free and single-rooted premolar teeth submerged (for a maximum of four weeks until use) in chloramine T trihydrate (Fluka Analytical, France) were retrieved from a university dental clinic. The crown of every tooth was decoronated at the cement-enamel junction (CEJ) using a ceramic cutting disk running at a speed of 100 rpm while being cooled by water (Struers, Glasgow, Scotland). With post drills (Parapost stainless drills, Coltène/Whaledent, Mahwah, NJ, USA), low speed hand piece and water cooling, post space preparations (Ø 1.7 mm) were made. An individual FRC post (1.5 mm, everStick, GC, Japan) was pre-cut (allowing for an approx. of 0.1–0.2 mm space around the entire circumference of the post) to the required length (8 mm) after drying the prepared canal. It’s length and compatibility were then confirmed by its insertion into the dried prepared root canal with a tweezer. After being removed from the canal, the post was shielded from light prior to luting. During this stage, the post had not yet undergone polymerization.

All teeth received the same adhesive treatment. After etching for 10 s using a 37% phosphoric acid etch-gel (Scotchbond, 3 M ESPE, USA), they were rinsed and gently air-dried. A disposable microbrush applicator was used in accordance with the manufacturer’s instructions to apply a dual-cure one-step adhesive system (G-Premio Bond and DCA, GC). Excess adhesive was removed by paper points and blowing air. The adhesive was light-cured for 60 s using a light-curing unit (Elipar TM S10, 3 M ESPE, Germany). The tooth surface was always in close proximity to the light-curing tip. The average light intensity of the light source, measured with a calibrator (Marc Resin Calibrator, BlueLight Analytics Inc., Canada) before the bonding procedure, was 1200 mW/cm^2^ and the wavelength was between 430 and 480 nm.

As control, conventional (particulate filled, PFC) dual-cure luting cement (G-CEM LinkForce A2, GC) was injected into the post space of half of the teeth (*n* = 10). With an ‘elongation tip’ for direct root canal application, the luting cement was applied using its own automix cartridge. The other half of specimens had light-cured SFRC (everX Flow, bulk shade, GC) as post luting material. For the purpose of removing extra cement out of the way of the post and preventing the creation of air bubbles, voids and other defects at the apical end of the canal, a cylinder-shaped stick (1.3 mm) was dipped into the fully filled post space. Then rounded unpolymerized post (everStick Post) was inserted into the canal. Half of posts from each group (*n* = 5) were soaked in bonding resin (Stick Resin, GC) for 5 min before insertion (post coating). The specimen was light cured through the FRC post for at least 60 s (Elipar TM S10).

After 48 h storage in water (37 °C), teeth were then horizontally sectioned perpendicular to the long axis of the post with a precision cutting saw (Struers, Glasgow, Scotland). These cross sections were 2 mm (± 0.1 mm) thick. From each tooth, three disks were obtained from the coronal and middle levels.

Four different groups were prepared, each having 10 specimens:

Group 1: Flowable SFRC as post luting material with no post coating

Group 2: Flowable SFRC as post luting material with post coating

Group 3: Conventional dual-cure PFC resin as post luting material with no post coating

Group 4: Conventional dual-cure PFC resin as post luting material with post coating

### Push-out test

**Figure 1.**
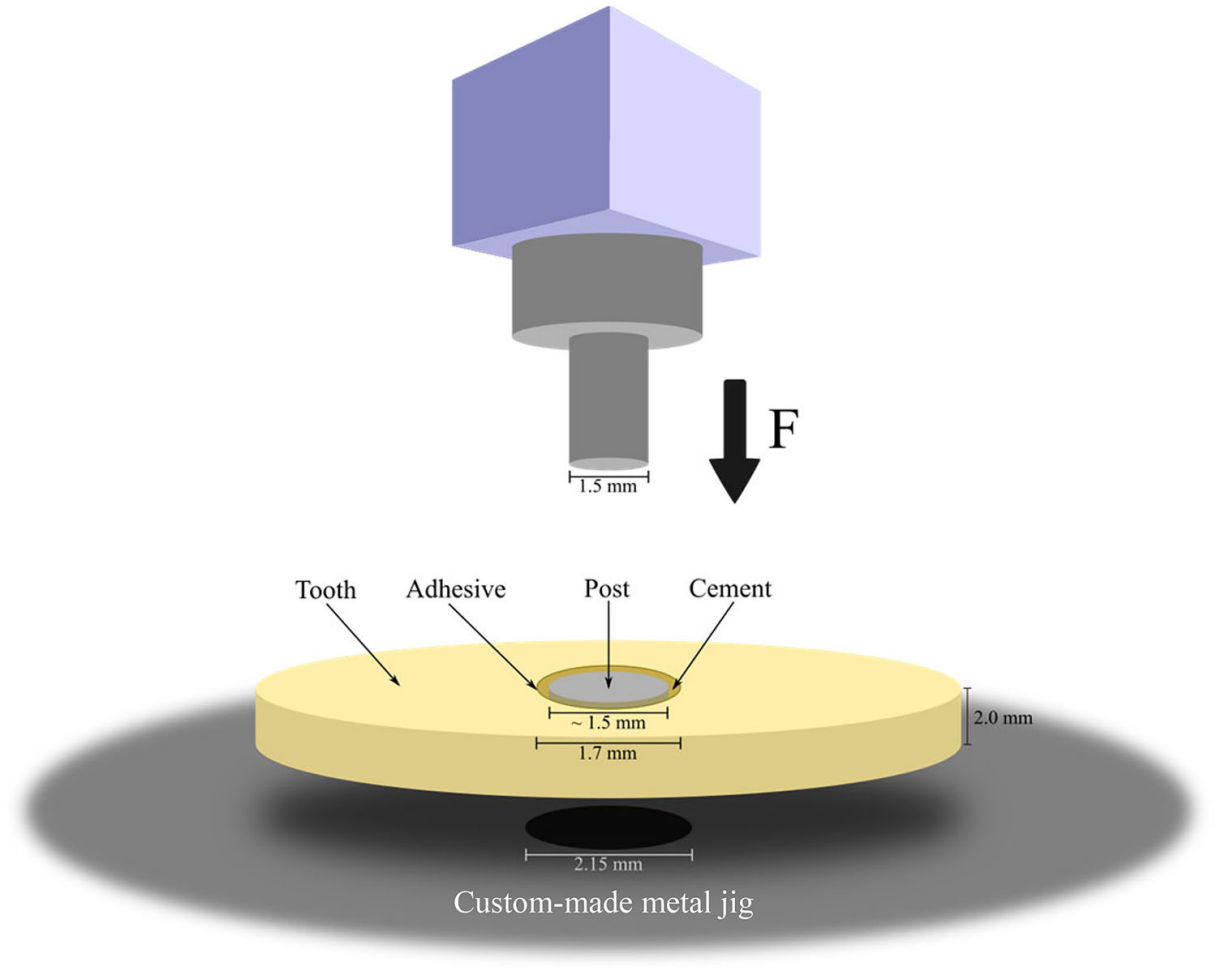
Schematic drawing of push-out test set-up.

Adhesion between FRC post and dentin was tested with a push-out test set-up in a universal testing machine (Model LRX, Lloyd Instruments Ltd., Fareham, England). The settings of the testing machine were: preload 3 N, preload speed 2 mm/min, extension rate 1 mm/min. In the test setting, a 1.5 mm flat cylinder end pushed specimen posts (Ø 1.5 mm) through a hole (custom-made metal jig) under the specimen (Figure 1). The hole was Ø 2.15 mm and a 4.3 mm deep cylinder was used being placed directly under the post. The machine stopped measuring when load had dropped to 40% from the peak value, as the post complex detached from the tooth. The data of maximum failure or debonding load (N) was collected using a computer program (Nexygen, Lloyd Instruments Ltd.) and converted into megapascal (MPa) using the following formula [16]:

$$\mathrm{Stress}\;\mathrm{at}\;\mathrm{Maximum}\;\mathrm{Load}=\sigma =\frac{F}{A}=\frac{\mathrm{Force}\;\mathrm{at}\;\mathrm{Maxixmum}\;\mathrm{Load}}{\pi \;\times \;\mathrm{post}\;\mathrm{diameter}\;\times \;\mathrm{height}}$$

Failure mode per specimen was analyzed using a stereomicroscope (Wild M3Z, Wild Heerbrugg, Switzerland) with different illumination angles and magnifications (6.5 and 15×) and categorized as interfacial failure between tooth and luting cement or post and luting cement.

### Microscope analysis of post-SFRC interface

The interface between FRC post and luting materials was analyzed using stereomicroscope with a magnification force of 15 and scanning electron microscopy (SEM, JSM 5500, Jeol Ltd., Tokyo, Japan). For light microscope analysis, teeth were sectioned vertically dividing the posts into halves (*n* = 2/per group). For SEM analysis, cross sectioned specimens were attached to SEM metal stands with conductive double-faced carbon adhesive tape (Nisshin EM Co., LTD., Japan) and left in an exicator for one day before they were gold coated (10 nm) in a sputter coater (BAL-TEC SCD 050 Sputter Coater, Balzers, Liechtenstein). SEM analysis was performed at an operating voltage of 15 kV, spot-size of 37 and working distance of 18 mm.

### Statistical analysis

Levene’s test was used to evaluate the assumption of equality of variances. After that, two-way ANOVA was used to determine the influence of the luting material and post coating influence on maximum stress (push-out strength) at maximum load. A significance level of 0.05 was used. Software for analysis was JMP®, Version 14.2.0 Pro. SAS Institute Inc., Cary, NC, 1989–2020.

## Results

**Figure 2.**
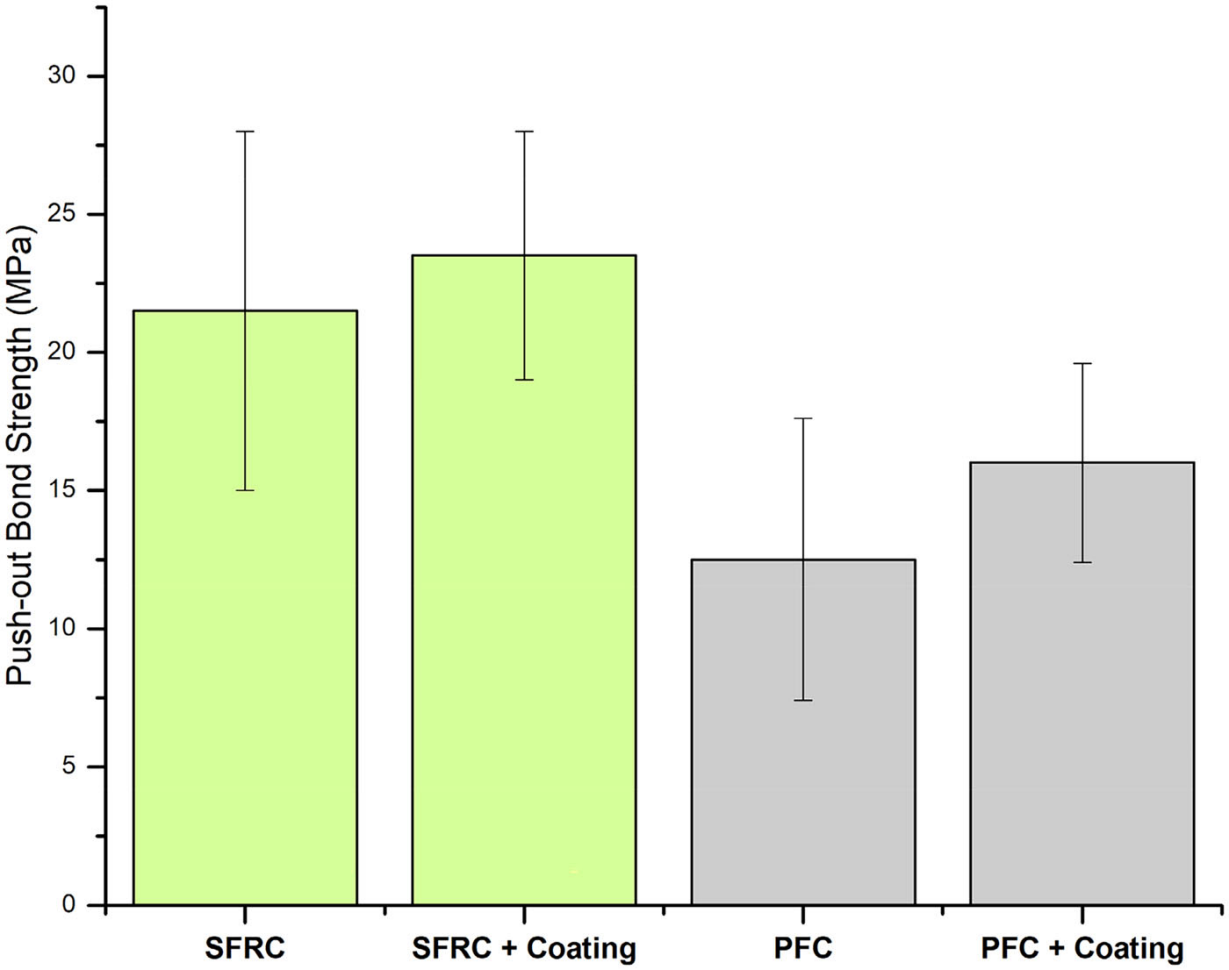
Graph illustrates means of push-out bond strength and standard deviation of FRC post luted to dentin either with SFRC or conventional dual-cure PFC resin. FRC post was either coated with bonding resin or not before luting.

The results of the push-out test are presented in Figure 2. ANOVA indicated that luting material type has a significant (*p* < .05) effect on FRC post adhesion. The highest bond strength values (23.5 MPa) were obtained after using SFRC (everX Flow) as post-luting material (*p* < .05). While, conventional dual-cure PFC resin showed the lowest values (12.5 MPa). Post coating didn’t show a statistically significant correlation to bonding strength (*p* = .102), neither between luting material type and post coating had a statistically significant correlation (*p* = .641). The variances were homogeneous and equal among groups, according to Levene’s test.

Regarding the failure modes, 11 (55%) SFRC specimens were broken between tooth and luting cement and 9 (45%) between luting cement and post. On the other hand, 7 (35%) PFC specimens were broken between tooth and cement and 13 (65%) between cement and post. In total, 18 (45%) had dislodged between tooth and cement and 22 (55%) between cement and post. When number of specimens having different dislodgement were compared between groups SFRC and PFC, statistically significant differences were not detected (Chi-Square Pearson, *p* = .204).

**Figure 3.**
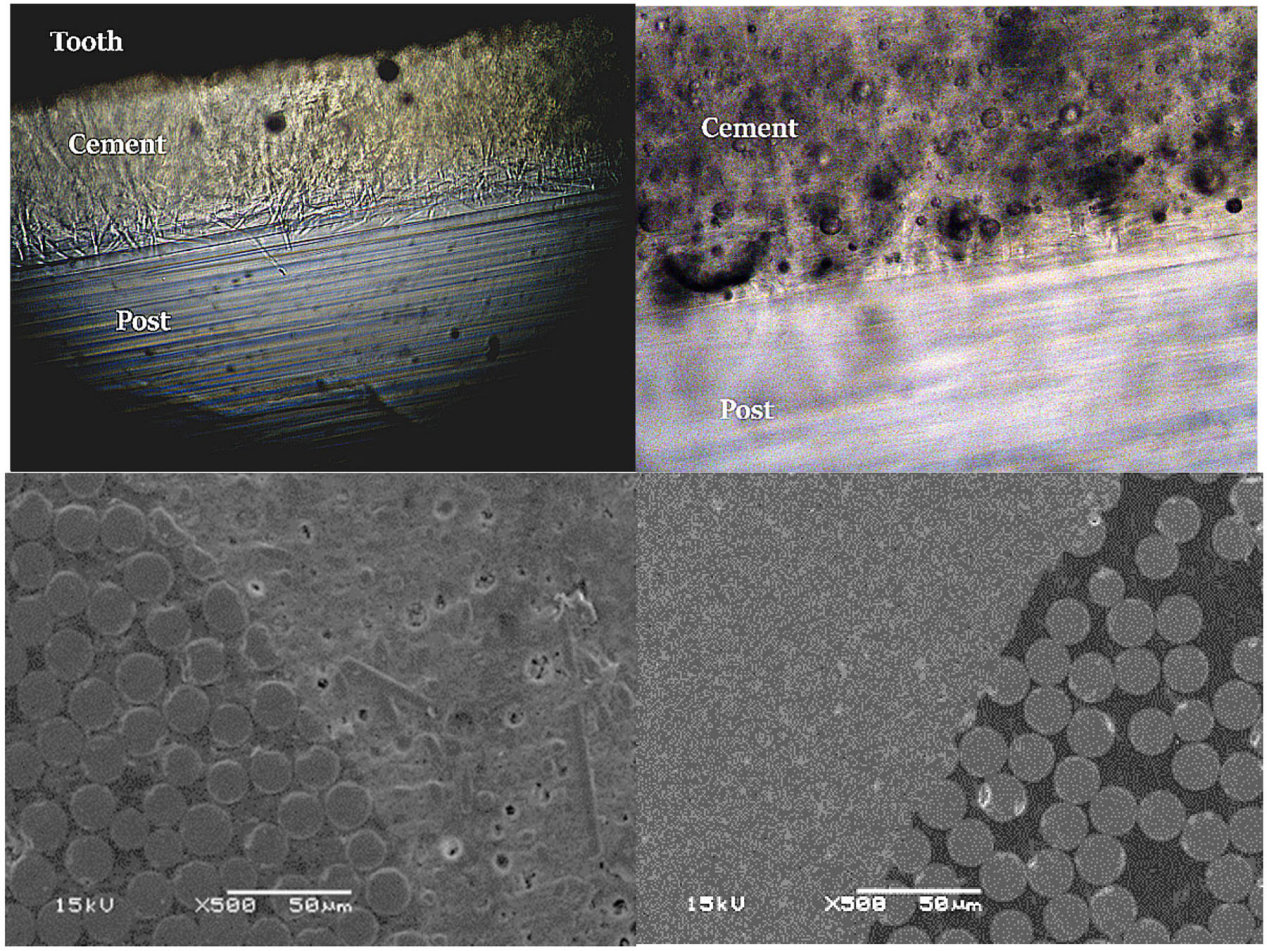
Light microscope and SEM images (500 × magnification) of the interface between FRC post and luting materials (SFRC:left side and PFC:right side).

Light microscope images (Figure 3) showed a pattern where short fibers of SFRC luting cement penetrate into FRC post. This happened in all specimens of SFRC groups regardless of the application of post coating. SEM images showed the bonding interface between fiber post and used luting materials, however, it was not able to show so clear penetration of luting material or fibers (Figure 3).

## Discussion

This pilot study was designed to evaluate the potential use of light-cured flowable SFRC as a post luting material. To our knowledge, this aspect has not been studied in existing literature. Flowable SFRC has been reported to have high fracture toughness and flexural properties compared to conventional dual-cured PFC resin [24]. Furthermore, recent studies showed the possibility of getting micro-mechanical interlocking between the monomer from successive PFC resin and protruded fibers from SFRC composite base [25,26]. As a result, we assume that the use of flowable SFRC as a luting material would be beneficial to improve the adhesion of FRC post to dentin.

Our results indicated that luting material type has influence on the push-out bonding strength of FRC post to dentin; therefore, the first null hypothesis was rejected. The findings of the present investigation (Figure 2) demonstrate that flowable SFRC could be used as post-luting material to achieve higher push-out bond strength values. These results can be explained from a range of perspectives. First, the presence of micro-mechanical interlocking between the protruding short fibers on the interface surfaces with FRC post (Figure 3, light microscope image). Particularly in the event of shear stress, this interlocking may affect bond strength values. Second, the SFRC's improved mechanical characteristics, particularly its fracture toughness, would increase it’s capacity to withstand shearing stresses [24,27]. In addition, randomly oriented fibers in SFRC have been demonstrated to affect the depth of the oxygen inhibition layer, which improves resin penetration at interfaces [28].

One of the key elements in load transfer is good adhesion between the luting material and the post, as well as the dentin and the luting material. Our results were supported by findings of some loading studies, where flowable SFRC was used as post-luting and core build-up material, in which fracture resistance of the post-core foundation showed the highest among all tested groups [21,23,29]. Authors stated that SFRC was tightly connected to the fiber post and root dentin, minimizing the drawbacks of using a weak link between them [21,29].

Despite the fact that there was little difference between groups regarding the failure modes, the use of SFRC demonstrated less adhesive failure at interface with FRC post compared to dual-cure PFC group. However, due to the semi interpenetrating polymer matrix structure (semi-IPN), the individually formed FRC posts demonstrated good bonding ability with luting cement and direct composite core restorations providing reliable surface retained applications [11,12,30]. In particular, when FRC posts were cured along with the luting cements after being inserted into the root canals, studies have shown that adhesive failures were predominately detected at the cement-dentin interfaces [12,30].

Applying bonding resin is reported to create a link between FRC post and luting cement and increase penetration depth in post and thus enhance adhesion by forming a solid adhesive interface [31]. Nevertheless, in this study, post coating with bonding resin did not have a significant effect on adhesion strength, neither between SFRC nor PFC specimens (Figure 2). Hence, the second hypothesis was accepted. This might be because the posts are already uncured, so it does not make a significant impact. Bonding resin could have marginal influence on uncured posts to either direction: either making a film over the post and hindering cement/fiber penetration or making a post’s surface more soluble.

Effective light transmission and scattering through the post is essential for optimal bonding and polymerization of FRC posts and luting cement [32]. In a simulated root canal, individually formed FRC posts with a semi-IPN polymer matrix demonstrated an appropriate level of monomer conversion and a tendency toward light conductivity [32]. However, it is uncertain if the light-cured SFRC will polymerize sufficiently inside the root canal. Earlier investigations by Lassila et al. and Frater et al. demonstrated that light-cured flowable SFRC material may be polymerized efficiently inside the root canal next to an individual FRC post, reaching just about the same microhardness levels as dual-cure material [22,23]. According to their approach for calculating microhardness, dual-cure PFC and flowable light-cure SFRC could both be utilized safely up to an 8 mm depth inside canals [23]. This could be traced back to multiple reasons, namely the light transmission of the FRC post [32], the transparency of the SFRC materials and the scattering of light by the short fibers [33]. However, having a dual-cure flowable SFRC as presented by Säilynoja and her colleagues would be an optimum and safe option for deeper canals [24]. From a clinical point of view, our results could shape the process of post cementation to a new direction, where flowable SFRC could be used as post core/luting material, without needing specific cement for the task.

The outcomes of our study may have been influenced by the inconsistency between the post space and post diameter. This discrepancy could have led to the creation of bubbles or gaps in the material, which is less likely to be seen in a thin and uniform layer of luting material [16,34].

The bond strength between a post and a tooth can be evaluated using different methods, such as conventional tensile testing on external root dentin [35] or on the post space surface using pullout [36] and push-out [11] techniques. The push-out method is preferred as it is more relevant to clinical situations, but there are concerns that this method may create a highly non-uniform stress at the adhesive interface when applied to the entire post or thick root segments [37]. Goracci et al. conducted a study to compare the accuracy of a microtensile technique with a push-out test for measuring the bond strength of fiber posts luted in post spaces. The authors found that the push-out test was more reliable than the microtensile technique as each specimen provided a useful measurement, and the data variability was low [38].

Though, the results of this study must be seen in the context of some limitations, some errors which can occur during specimen preparation where in which unpolymerized post might not perfectly round when inserted, although they were rolled to be round. This would create an error in calculations and testing. Also, posts were not perfectly in the middle of the post space, but at the push-out test, the pushing head was placed on posts. It should be noted that the mechanical properties and conversion rate of the luting material can impact the stress distribution and failure modes at the adhesive interface, which can ultimately affect the push-out force and bond strength values.

Another limitation of this study is that we did not assess the variation in push-out bond strength between different root sections, such as the coronal and middle levels. However, numerous studies in the literature have indicated that bonding at the coronal level of the root canal appears to be more reliable than bonding at the middle or apical level [39,40].

## Conclusions

The use of flowable SFRC as luting material with individually formed FRC post proved to be promising method to improve the interface adhesion.
